# *N*-Alkyl derivatives of diosgenyl 2-amino-2-deoxy-β-D-glucopyranoside; synthesis and antimicrobial activity

**DOI:** 10.3762/bjoc.11.97

**Published:** 2015-05-22

**Authors:** Agata Walczewska, Daria Grzywacz, Dorota Bednarczyk, Małgorzata Dawgul, Andrzej Nowacki, Wojciech Kamysz, Beata Liberek, Henryk Myszka

**Affiliations:** 1 Department of Chemistry, University of Gdańsk, Wita Stwosza 63, 80-308 Gdańsk, Poland; 2 Department of Pharmacy, Medical University of Gdańsk, Hallera 107, 80-416 Gdańsk, Poland

**Keywords:** antimicrobial activities, D-glucosamine, diosgenin glycosylation, *N*-alkylation

## Abstract

Diosgenyl 2-amino-2-deoxy-β-D-glucopyranoside is a synthetic saponin exhibiting attractive pharmacological properties. Different pathways tested by us to obtain this glycoside are summarized here. Moreover, the synthesis of *N*-alkyl and *N*,*N*-dialkyl derivatives of the glucopyranoside is presented. Evaluation of antibacterial and antifungal activities of these derivatives indicates that they have no inhibitory activity against Gram-negative bacteria, whereas many of the tested *N*-alkyl saponins were found to inhibit the growth of Gram-positive bacteria and human pathogenic fungi.

## Introduction

Saponins are a group of steroid or triterpenoid glycosides, widely distributed in the plant kingdom [1]. Saponins are characteristic by their foaming properties in aqueous solution, causing them to be used as detergents, surfactants and emulsifiers. Moreover, they display a wide range of pharmacological activities, including antifungal, antiparasitic, antiinflammatory, antibacterial, and antitumor activities [2–5]. No wonder, saponins have been evaluated as vaccine adjuvants [6]. Despite the fact that thousands of homogeneous saponins have been characterized, new types of saponins are regularly isolated from nature and their biological activities are evaluated [7–11]. The yields of homogenous saponins isolated from natural sources are rather low. Therefore, chemical synthesis of saponins have been investigated [12–22] as well as the evaluation of their antitumor activities [23–27].

Naturally-occurring diosgenyl glycosides are the most abundant steroidal saponins. They have been continuously synthesized [28–32] and their cardiovascular, antifungal, anticancer [33–36] and antithrombotic activities [37] have been investigated. Gelation ability of the pentose derivatized diosgenyl saponins have also been reported [38]. In the family of diosgenyl β-glycosides D-glucopyranose is the first sugar attached to the diosgenin. Very often this D-glucopyranose is substituted with α-L-rhamnopyranose at 2-OH and other sugars at 4-OH. The change of D-glucopyranose into 2-amino-2-deoxy-D-glucopyranose provides diosgenyl 2-amino-2-deoxy-β-D-glucopyranoside (**7**), a synthetic saponin, first reported by us [39]. It was also demonstrated that diosgenyl 2-amino-2-deoxy-β-D-glucopyranoside hydrochloride increases the number of apoptotic B cells, in combination with cladribine (2-CdA), which were isolated from chronic lymphotic leukemia (B-CLL) patients [40]. The presence of the amine group in this promising antitumor compound creates the opportunity to synthesize new analogues with an enhanced activity. In this way, many of the *N*-acyl [41–43] as well as urea and thiosemicarbazone [44] analogues of **7** have been obtained and evaluated. Their characteristic feature is that their amine group is bound with the carbonyl or thiocarbonyl group. Such analogues are much more lipophilic, but also less basic than the parent saponin **7**. In this paper, for the first time, the synthesis and antimicrobial activity of the *N*-alkyl derivatives of diosgenyl 2-amino-2-deoxy-β-D-glucopyranoside (**7**) are presented. From the chemical point of view, the alkyl group has quite different properties than the acyl group. First, alkylation does not change significantly the basicity of the parent amine group. Thus, the ability to bind protons by the parent compound and its analogue should be comparable. Second, the *N*-alkylamine group, similarly to the amine group, is able to work as a hydrogen bond acceptor. On the other hand, alkylation improves lipophilicity of the compound, which may be crucial for its biological activity.

At the beginning, our experiences concerning the synthesis of the parent diosgenyl 2-amino-2-deoxy-β-D-glucopyranoside (**7**) are summarized and presented. We have studied different glycosyl donors, different amine group protections, different solvents and promoters to find the best way to obtain **7**. The presented compilation is informative for all those interested in the glycosidation of 2-amino-2-deoxy sugars.

## Results and Discussion

### Chemistry

**Scheme 1:**
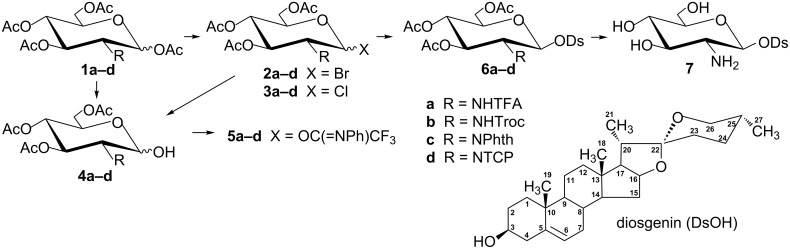
Reagents used for the synthesis of diosgenyl 2-amino-2-deoxy-β-D-glucopyranoside (**7**).

To synthesize the parent diosgenyl 2-amino-2-deoxy-β-D-glucopyranoside (**7**), the *O*-acetylated bromides **2a**–**d**, chlorides **3a**–**d** and (*N*-phenyl)trifluoroacetimidates **5a**–**d**, α or β anomers, were examined (Scheme 1). These glycosyl donors were *N*-protected with trifluoroacetyl (TFA, **a**), 2,2,2-trichloroethoxycarbonyl (Troc, **b**), phthaloyl (Phth, **c**), and tetrachlorophthaloyl (TCP, **d**) groups, respectively. Applications of bromides **2a** and **2d** [40] and chlorides **3a–d** [45] were previously reported. Here, applications of the two remaining bromides **2b** and **2c** as well as (*N*-phenyl)trifluoroacetimidates **5a–d** are demonstrated. To synthesize bromide **2b** we used a procedure described by Ellervik and Magnusson for other glycosylations [46]. However, the bromide obtained by them was a mixture of α and β anomers whereas **2b** is a pure α anomer (*J*_1,2_ 4,0 Hz). Bromide **2b**, identified as the α anomer, was also synthesized by Higashi et al., but in a slightly different manner [47]. Bromide **2c** (α + β) was synthesized analogously to **2b**. Synthesis of (*N*-phenyl)trifluoroacetimidates **5a–d** demands removal of the acetyl groups from the anomeric hydroxy groups in **1a–d**. It was done with ethylenediamine in a mixture with acetic acid in THF, a procedure adopted from Zhang and Kováč [48]. This selective 1-*O*-deacetylation turned out to be very effective for **1a**, **1b**, and **1c** (97%, 84%, and 90%, respectively). However, this was quite ineffective for **1d** (36%). Therefore, 1-*O*-deacetylation of the latter was carried out via hydrolysis (Ag_2_CO_3_, acetone/H_2_O 2:1) of bromide **2d** (73% yield) or chloride **3d** (72% yield). (*N*-Phenyl)trifluoroacetimidates **5a–d** were synthesized in reaction of the respective *N*-protected 3,4,6-tri-*O*-acetyl-D-glucosamines **4a–d** with *N*-phenyltrifluoroacetimidoyl chloride, according to a procedure proposed by Yu and Tao for 1-hydroxy derivatives of D-glucose and L-rhamnose [49].

Glycosylation of diosgenin with twelve different derivatives of D-glucosamine (**2a–d**, **3a–d**, and **5a–d**), was examined using “normal” and “reverse” procedures [50] (Table 1). In the “normal” procedure, the promoter (silver triflate or trimethylsilyl triflate) was added to the solution of diosgenin and the respective glycosyl donor. In the “reverse” procedure, the respective glycosyl donor was added to the solution of diosgenin and the promoter. Diosgenin glycosylations were carried out in dichloromethane or/and in a mixture of dichloromethane and diethyl ether. The results summarized in Table 1 indicate that the “reverse” procedure is much more effective than the “normal” procedure. Running of the diosgenin glycosylation also depends on the kind of the solvent used. It is particularly important when bromide **2a** is used as a glycosyl donor. Reaction of **2a** with diosgenin conducted by the “reverse” procedure in the CH_2_Cl_2_/Et_2_O mixture leads to glycoside **6a** in 77% yield. The same procedure applied in CH_2_Cl_2_ gives no glycoside. Similarly, reaction of **2b** with diosgenin conducted by the “reverse” procedure in the CH_2_Cl_2_/Et_2_O mixture gives glycoside **6b** in an excellent 98% yield. In turn, bromides with the phthaloyl protections of the amine group (**2c** and **2d**) react more effectively with diosgenin when the reagents are dissolved solely in CH_2_Cl_2_. A comparison of the efficiency of the glycosyl donors indicates that the yields of diosgenin glycosylation with bromides **2a**, **2b** and **2d** are higher than those with analogous chlorides **3a**, **3b** and **3d**. However, reactivity of chloride **3c** is stronger than that of analogous bromide **2c**. (*N*-Phenyl)trifluoroacetimidates (**5a**–**d**) seem to be quite effective glycosyl donors. However their comparison with halogens **2a–d** and **3a–d** is encumbered since the glycosylation conditions were different for **5a–d**. The *N*-trifluoroacetyl-protected bromide **2a** is the less reactive among the bromides **2a–d**; the remaining bromides react similarly. The same refers to chlorides **3a–d**. In the case of (*N*-phenyl)trifluoroacetimidates **4a**–**d**, the least efficient is **4d** with the tetrachlorophthaloyl protection of the amine group; the remaining (*N*-phenyl)trifluoroacetimidates react similarly. In the experimental section (see Supporting Information File 1), the best procedures for each glycosyl donor are presented. Finally, diosgenyl 2-amino-2-deoxy-β-D-glucopyranose (**7**) was obtained by complete deprotections of **6a–d**, as previously reported [45].

**Table 1:** Procedures and results concerning diosgenin glycosylation.

Entry	Procedure	Glycosyl donor	Solvent	Promoter	Product	Yield (%)

1	normal	**2a** (α)	CH_2_Cl_2_/Et_2_O	AgOTf	**6a**	30
2	reverse	**2a** (α)	CH_2_Cl_2_/Et_2_O	AgOTf	**6a**	77
3	reverse	**2a** (α)	CH_2_Cl_2_	AgOTf	―	―
4	reverse	**2b** (α)	CH_2_Cl_2_/Et_2_O	AgOTf	**6b**	98
5	normal	**2c** (α + β)	CH_2_Cl_2_/Et_2_O	AgOTf	**6c**	51
6	reverse	**2c** (α + β)	CH_2_Cl_2_/Et_2_O	AgOTf	**6c**	55
7	reverse	**2c** (α + β)	CH_2_Cl_2_	AgOTf	**6c**	90
8	normal	**2d** (α + β)	CH_2_Cl_2_/Et_2_O	AgOTf	**6d**	73
9	reverse	**2d** (α + β)	CH_2_Cl_2_	AgOTf	**6d**	93
10	reverse	**3a** (α)	CH_2_Cl_2_/Et_2_O	AgOTf	**6a**	69
11	reverse	**3b** (α)	CH_2_Cl_2_/Et_2_O	AgOTf	**6b**	86
12	reverse	**3c** (β)	CH_2_Cl_2_	AgOTf	**6c**	99
13	reverse	**3d** (β)	CH_2_Cl_2_	AgOTf	**6d**	87
14	normal	**5a** (α + β)	CH_2_Cl_2_	TMSOTf	**6a**	85
15	normal	**5b** (α + β)	CH_2_Cl_2_	TMSOTf	**6b**	81
16	normal	**5c** (β)	CH_2_Cl_2_	TMSOTf	**6c**	83
17	normal	**5d** (β)	CH_2_Cl_2_	TMSOTf	**6d**	52

**Scheme 2:**
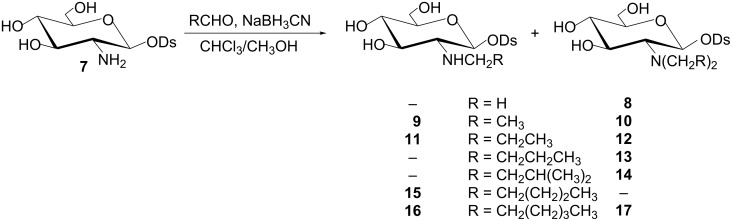
*N*-Alkylation of diosgenyl 2-amino-2-deoxy-β-D-glucopyranoside (**7**).

To obtain *N*-alkyl derivatives of diosgenyl 2-amino-2-deoxy-β-D-glucopyranoside (**7**), a method called “reductive alkylation of amines” was chosen. This method was previously successfully used to prepare *N*-alkyl derivatives of 1,3,4,6-tetra-*O*-acetyl-2-amino-2-deoxy-D-glucose [51–52]. Thus, reductive alkylation of **7** with a 1.2 molar excess of the appropriate aldehyde and a twofold molar excess of NaBH_3_CN provided mono- (**9**, **11**, **15**, **16**) and dialkylated products (**8**, **10**, **12**–**14**, **17**), solely or as mixtures (Scheme 2). The respective mixtures of mono- and dialkylated products were separated. Structures of the *N*-alkylated derivatives of **7** were confirmed by the NMR (^1^H and ^13^C) spectroscopy and mass spectrometry (see Supporting Information File 1). All of them, similarly to the parent diosgenyl glycoside (**7**), adopt the ^4^*C*_1_ conformation, as demonstrated by the *J*_1,2_ ≈ 8 Hz, *J*_2,3_ ≈ *J*_3,4_ ≈ *J*_4,5_ ≈ 9–10 Hz coupling constants.

### Evaluation of antimicrobial activity

The *N*-alkyl derivatives of diosgenyl 2-amino-2-deoxy-β-D-glucopyranosides **8**–**15** and **17** were tested for their antibacterial and antifungal in vitro activity against 5 strains of Gram-negative bacteria, 5 strains of Gram-positive bacteria, and 3 strains of human pathogenic fungi. Respective minimum inhibitory concentration (MIC) values determined by a serial dilution microplate method according to the guidelines of the Clinical and Laboratory Standards Institute (CLSI) for **8**–**15** and **17** as well as for **7** hydrochloride are summarized in Table 2 and Table 3. The latter was added as the reference since its high in vitro activities and in vivo efficacy were proved [53].

The determined MIC values indicate that compounds explored have rather poor if any inhibitory activity against the Gram-negative bacteria, such as *Escherichia coli*, *Klebsiella pneumoniae*, *Proteus mirabilis*, *Proteus vulgaris* and *Pseudomonas aeruginosa*. In contrast, almost all the tested *N*-alkyl saponins were found to inhibit the growth of the Gram-positive bacteria (Table 2). Among the tested compounds the most active was diosgenyl 2-deoxy-2-ethylamino-β-D-glucopyranoside (**9**) with MIC of 0.5, 1, 2, and 8 μg/mL against *Staphylococcus epidermidis*, *Enterococcus faecalis*, *Staphylococcus aureus*, and *Rhodococcus equi*, respectively. Also the *N*-propyl derivative **11** was found to be active against *Enterococcus faecalis*, *Staphylococcus epidermidis*, and *Staphylococcus aureus* with MIC of 1, 1, and 8 μg/mL, respectively. Importantly, both **9** and **11** exhibit a stronger inhibitory effectivity than diosgenyl 2-deoxy-2-amino-β-D-glucopyranoside hydrochloride (**7**·HCl), which was found to be very active alone and in combination with daptomycin and vancomycin against Gram-positive cocci [53]. The *N,N*-dialkyl derivatives **12**, **13** and **14** act against the Gram-positive bacteria more efficiently or similarly to **7** hydrochloride. In turn, *N*-pentyl (**15**) and *N*,*N*-dihexyl (**17**) compounds are completely inactive with respect to all the tested strains of the Gram-positive bacteria. The results presented indicate that both elongation of the alkyl group as well as addition of another alkyl group are rather ineffective from the standpoint of the inhibitory activity towards the Gram-positive bacteria. Such findings are probably due to the lower solubility of the compounds with longer *N*-alkyl groups or to the micelle formation.

**Table 2:** Minimum inhibitory concentration (MIC) [μg/mL] for **8**–**15** and **17** against the Gram-positive bacteria.

Comp.	*Bacillus subtilis*	*Enterococcus faecalis*	*Rhodococcus equi*	*Staphylococcus aureus*	*Staphylococcus epidermidis*

**7·**HCl	8	16	16	16	16
**8**	8	32	64	32	64
**9**	64	1	8	2	0.5
**10**	32	32	16	32	32
**11**	64	1	32	8	1
**12**	16	8	16	8	32
**13**	16	8	16	8	8
**14**	32	4	8	16	32
**15**	>1024	>1024	512	1024	1024
**17**	64	64	512	128	256

Studies on the activity of the synthesized *N*-alkyl derivatives of diosgenyl 2-deoxy-2-amino-β-D-glucopyranoside (**8**–**15** and **17**) against 3 strains of the human pathogenic fungi (Table 3) indicate that the growth of *Candida tropicalis* is the most efficiently inhibited by the reference compound **7**·HCl with MIC of 0.5 μg/mL. A slightly lower activity was exhibited by *N*,*N*-diethyl derivative **10** (MIC 1 μg/mL), *N*,*N*-dimethyl derivative **8** (MIC 2 μg/mL), and *N*,*N*-dipropyl derivative **12** (MIC 4 μg/mL). Compounds with the longer alkyl chains (**13**–**15**, **17**) show very weak inhibitory activity against *Candida tropicalis*.

**Table 3:** Minimum inhibitory concentration (MIC) [μg/mL] for **8**–**15** and **17** against human pathogenic fungi.

Comp.	*Candida tropicalis*	*Candida albicans*	*Aspergillus niger*

**7**·HCl	0.5	2	64
**8**	2	2	64
**9**	—^a^	16	8
**10**	1	2	128
**11**	—^a^	8	8
**12**	4	4	64
**13**	128	8	16
**14**	32	16	256
**15**	64	128	128
**17**	128	64	128

^a^Not determined.

The results presented for *Candida albicans* resemble those obtained for *Candida tropicalis*. The growth of this strain of fungi is inhibited at the lowest concentrations by **7**·HCl and equally well by **8**, **10**, and **12** (MIC 2 μg/mL for each compound mentioned). Evidently, short dialkyl derivatives (**8**, **10**, **12**) are more effective against the tested fungi than analogous monoalkyl derivatives (**9**, **11**), longer monoalkyl (**15**) and dialkyl derivatives (**13**, **14**, **17**).

Among the tested strains of fungi, *Aspergillus niger* turned out to be the least susceptible to the *N*-alkyl derivatives of diosgenyl 2-deoxy-2-amino-β-D-glucopyranoside (**8**–**15** and **17**). It is worth notice that *N*-ethyl (**9**) and *N*-propyl (**11**) derivatives reveal much better activity than the reference **7**·HCl (MIC 8, 8, and 64 μg/mL, respectively). Also *N*,*N*-dibutyl derivative (**13**) with MIC of 16 μg/mL inhibits the growth of *Aspergillus niger* at lower concentrations in comparison to that of **7**·HCl.

## Conclusion

Different pathways leading to diosgenyl 2-amino-2-deoxy-β-D-glucopyranoside and several *N*-alkyl derivatives are reported. Investigations of their antimicrobial activity indicate that *N*-ethyl and *N*-propyl derivatives exhibit stronger activity against Gram-positive bacteria than the parent diosgenyl 2-deoxy-2-amino-β-D-glucopyranoside hydrochloride.

## Supporting Information

File 1 Experimental details for the preparation of compounds **2b**, **2c**, **4a–d**, **5a–d**, **6a–d**, **8**–**17**, corresponding characterization data and information on the way of determination of minimum inhibitory concentration.
