# Supplementary material for: N-Alkyl derivatives of diosgenyl 2-amino-2-deoxy-β-D-glucopyranoside; synthesis and antimicrobial activity
Source: Beilstein J Org Chem. 2015 May 22;11:869–74. doi: 10.3762/bjoc.11.97 (PMC4464337; doi:10.3762/bjoc.11.97)
Supplement: File 1 — Experimental details for the preparation of compounds 2b, 2c, 4a–d, 5a–d, 6a–d, 8–17, corresponding characterization data and information on the way of determination of minimum inhibitory concentration. [file Beilstein_J_Org_Chem-11-869-s001.pdf]

Supporting Information

for

N-Alkyl derivatives of diosgenyl 2-amino-2-deoxy-β-D-glucopyranoside; synthesis and antimicrobial activity

Agata Walczewska[1], Daria Grzywacz[1], Dorota Bednarczyk[1], Małgorzata Dawgul[2], Andrzej Nowacki[1], Wojciech Kamysz[2], Beata Liberek*[,§,1], Henryk Myszka[1]

Address: [1]Department of Chemistry, University of Gdańsk, Wita Stwosza 63, 80-308 Gdańsk, Poland and [2]Department of Pharmacy, Medical University of Gdańsk, Hallera 107, 80-416 Gdańsk, Poland

Email: Beata Liberek*- beata.liberek@ug.edu.pl

*Corresponding author.

[§]Tel.: + 48 58 5235071; Fax: + 48-58-5235012;

Experimental details for the preparation of compounds 2b, 2c, 4a–d, 5a–d, 6a–d, 8–17, corresponding characterization data and information on the way of determination of minimum inhibitory concentration.

Table of contents

Experimental details …………………………………………………………………..…… S2

Determination of minimum inhibitory concentration …………………………………….. S17

References …………………………………………………………………………………… S18

Experimental

General methods

Solvents and chemical reagents were purchased and used without further purification. Melting points are uncorrected. The IR spectra were recorded as Nujol mulls with a Bruker IFS 66 spectrophotometer. The ^{1}H and ^{13}C NMR spectra were recorded in $CDCl_3$ or a mixture of $CDCl_3$:CD_3OD (1:1, v/v) with internal Me_4Si on a Varian Mercury 400 MHz instrument (400.49/100.70 MHz). Positive-ion mode MALDI-TOF mass spectra were obtained using a Bruker Biflex III spectrometer with 4-cyano-4-hydroxycinnamic acid or 2,5-dihydroxy-benzoic acid matrixes. The optical rotations were determined at rt on a Perkin-Elmer polarimeter in a 1-dm tube at the D line of sodium using $CHCl_3$ or a mixture of $CHCl_3$:CH_3OH (1:1, v/v) as the solvents. Elemental analyses were performed on a Carlo Erba EA 1108 analyzer. Thin-layer chromatography (TLC) was performed on aluminium plates coated with E. Merck Kieselgel 60 F_{254} using the following eluent systems (v/v): A, 2:1 toluene : AcOEt; B, 10:1 $CHCl_3$: Et_2O; C, 4:1 CCl_4 : acetone; D, 7:1 toluene : AcOEt; E, 6:1 toluene : AcOEt, F, 5:1 toluene : AcOEt; G, 4:1 $CHCl_3$: MeOH; H, 2:1 CCl_4 : acetone; I, 7:1 $CHCl_3$: MeOH; J, 5:1 $CHCl_3$: MeOH. Column chromatography was performed on MN Kieselgel 60 (<0.8 mm) with one of the above listed eluent systems. For the detection of compounds the dry plates were sprayed with a 5% aqueous sulfuric acid solution and then heated at 150 °C.

3,4,6-Tri-*O*-acetyl-2-deoxy-2-(2,2,2-trichloroethoxycarbonylamino)-α-D-gluco-pyranosyl bromide (**2b**)

To a solution of **1b** [1] (0.6 g, 1.15 mmol) in glacial acetic acid (2 mL) and acetic anhydride (0.4 mL), a 45% solution of HBr in acetic acid (3 mL) was added. The mixture was stirred at rt. After 1 h the mixture was diluted with $CHCl_3$ (40 mL), washed with H_2O (2 x 10 mL), satd

aq $NaHCO_3$ (2 x 10 mL) and again H_2O (20 mL). Then it was dried over $MgSO_4$ and concentrated. This afforded **2b** (0.6 g, white foam, 96%): $[\alpha]_D^{20}$ 135; R_f 0.60 (solvent A); IR: ν 3345 (N-H), 1751 (C=O/Ac and C=O/Troc), 1538 cm^{-1} (N-H, C-N); ^1H NMR (400 MHz, $CDCl_3$): in accordance with literature data [2]; ^{13}C NMR (100 MHz, $CDCl_3$): *δ* 171.18, 170.71, 169.43 (3 x C=O/Ac), 154.23 (C=O/Troc), 91.19 (C-1), 74.94 (CH_2/Troc), 72.88 (C-5), 70.87 (C-3), 67.03 (C-4), 61.15 (C-6), 55.80 (C-2), 20.86, 20.79, 20.74 (3 x CH_3); MALDI-TOF-MS: *m/z* 544.2 $(M+H)^+$.

3,4,6-Tri-*O*-acetyl-2-deoxy-2-phthalimido-α,β-D-glucopyranosyl bromide (**2c**)

To a solution of **1c** [1] (0.58 g, 1.2 mmol) in glacial acetic acid (2 mL) and acetic anhydride (0.55 mL), a 45% solution of HBr in acetic acid (2 mL) was added. The mixture was stirred at rt. After 1 h the mixture was diluted with $CHCl_3$ (40 mL), washed with H_2O (2 x 10 mL), satd aq $NaHCO_3$ (2 x 10 mL) and again H_2O (20 mL). Then it was dried over $MgSO_4$ and concentrated. This afforded **2c** (0.6 g, white foam, 98%): R_f 0.5 (solvent A); ^1H NMR (400 MHz, $CDCl_3$) (α): *δ* 7.88 (m, 2 H, Phth), 7.78 (m, 2 H, Phth), 6.67 (dd, 1 H, $J_{2,3}$ 11.6, $J_{3,4}$ 9.2 Hz, H-3), 6.58 (d, 1 H, $J_{1,2}$ 3.2 Hz, H-1), 5.17 (t, 1 H, $J_{4,5}$ = $J_{3,4}$ 9.2 Hz, H-4), 4.70 (dd, 1 H, $J_{2,3}$ 11.6, $J_{1,2}$ 3.2 Hz, H-2), 4.35 (m, 2 H, H-5, H-6), 4.18 (dd, 1 H, $J_{5,6'}$ 2.4, $J_{6,6'}$ 12.4 Hz, H-6'); (β): *δ* 7.88 (m, 2 H, Phth), 7.78 (m, 2 H, Phth), 6.42 (d, 1 H, $J_{1,2}$ 9.6 Hz, H-1), 5.77 (dd, 1 H, $J_{2,3}$ 10.4, $J_{3,4}$ 9.2 Hz, H-3), 5.27 (dd, 1 H, $J_{3,4}$ 9.2, $J_{4,5}$ 10.0 Hz, H-4), 4.63 (dd, 1 H, $J_{1,2}$ 9.6, $J_{2,3}$ 10.4 Hz, H-2), 4.34 (dd, 1 H, $J_{5,6}$ 4.8, $J_{6,6'}$ 12.4 Hz, H-6), 4.21 (dd, 1 H, $J_{5,6'}$ 2.4, $J_{6,6'}$ 12.4 Hz, H-6'), 3.98 (m, 1 H, H-5); (α+β): 2.14, 2.12, 2.04, 2.09, 1.91, 1.87 (6 s, 18 H, 6 x OAc); ^{13}C NMR (100 MHz, $CDCl_3$) (α): *δ* 87.37 (C-1), 72.61 (C-5), 69.00 (C-4), 67.75 (C-3), 61.15 (C-6), 56.40 (C-2); (β): *δ* 77.55 (C-1), 76.91 (C-5), 70.75 (C-3), 68.29 (C-4), 61.85 (C-6), 58.29 (C-2); (α+β): *δ* 170.77, 170.65, 170.12, 170.08, 169.45, 169.30 (10 x C=O), 134.75, 124.03 (Ph), 20.89, 20.88, 20.83, 20.71, 20.51 (6 x CH_3).

3,4,6-Tri-*O*-acetyl-2-deoxy-2-trifluoroacetamido-α-D-glucopyranose (**4a**)

Ethylenediamine (0.088 mL, 1.35 mmol) was added to a solution of **1a** (0.5 g, 1.13 mmol) in tetrahydrofuran (28 mL) and glacial acetic acid (88 μL, 1.56 mmol). After stirring for 15 h H_2O (15 mL) was added to the reaction mixture. Then it was extracted with CH_2Cl_2 (3 x 30 mL). The organic extracts were combined, washed with 3% aq HCl, satd aq $NaHCO_3$ (2 x 30 mL) and H_2O (20 mL), dried over $MgSO_4$ and concentrated. This gave **4a** (0.44 g, 97%): mp 165-168 ^{o}C, lit.[3] mp 174 ^{o}C; $[\alpha]_D^{20}$ +19 (*c* 0.5, $CHCl_3$), lit.[3] $[\alpha]_D^{20}$ +20 (*c* 0.5, $CHCl_3$); R_f 0.3 (solvent A); spectroscopic analysis in accordance with literature data [3]; Anal. Calcd for $C_{14}H_{18}O_9NF_3$: C, 41.90; H, 4.52; N, 3.49. Found: C, 42.06; H, 4.58; N, 3.44.

3,4,6-Tri-*O*-acetyl-2-deoxy-2,2,2-trichloroethoxycarbonylamino-D-glucopyranose (**4b**)

This was synthesized analogously to **4a**. The following amounts of the substrates were used: **1b** (0.8 g, 1.53 mmol), ethylenediamine (0.12 mL, 1.84 mmol), tetrahydrofuran (39 mL) and glacial acetic acid (0.12 mL, 2.13 mmol). The crude white product (0.73 g) was crystallized from AcOEt/hexane to afford **4b** (0.62 g, white powder, 84%): R_f 0.32 (α) and 0.17 (β) (solvent A); ^{1}H NMR (400 MHz, $CDCl_3$) (α): δ 5.45 (d, 1 H, NH), 5.34 (dd, 1 H, $J_{2,3}$ 9.6, $J_{3,4}$ 10.4 Hz, H-3), 5.32 (d, 1 H, $J_{1,2}$ 3.2 Hz, H-1), 5.13 (dd, 1 H, $J_{4,5}$ 9.6, $J_{3,4}$ 10.4 Hz, H-4), 4.80 (d, 1 H, CH_ATroc), 4.64 (d, 1 H, CH_BTroc), 4.26-4.22 (m, 2 H, H-5, H-6'), 4.15 (dd, 1 H, $J_{5,6}$ 4.0, $J_{6,6'}$ 14.0, H-6), 4.05 (dt, 1 H, $J_{1,2}$ 3.2 Hz, $J_{2,3} = J_{2,NH}$ 9.6 Hz, H-2), 3.63 (d, 1 H, OH), 2.11, 2.05, 2.02 (3 s, 9 H, 3 x OAc); ^{13}C NMR (100 MHz, $CDCl_3$) (α): δ 171.27, 170.11, 169.71 (3 x ester C=O), 154.47 (Troc C=O), 92.02 (C-1), 74.82 (Troc CH_2), 70.95 (C-3), 68.51 (C-4), 67.95 (C-5), 62.25 (C-6), 54.38 (C-2), 20.99, 20.92, 20.85 (3 x acetyl CH_3); Anal. Calcd for $C_{15}H_{20}O_{10}NCl_3$: C, 37.48; H, 4.19; N, 2.91. Found: C, 37.45; H, 4.20; N, 2.90.

3,4,6-Tri-*O*-acetyl-2-deoxy-2-phthalimido-β-D-glucopyranose (**4c**)

This was synthesized analogously to **4b**. The following amounts of the substrates were used: **1c** (0.27 g, 1.53 mmol), ethylenediamine (0.12 mL, 1.84 mmol), tetrahydrofuran (39 mL) and glacial acetic acid (0.12 mL, 2.13 mmol). This afforded **4c** (0.23 g, 90%): mp 170-172 °C; $[\alpha]_D^{20}$ +67 (*c* 0.5, $CHCl_3$); R_f 0.28 (solvent A); IR: ν 3522 (O-H), 1745, 1785 (C=O/OAc), 1713 cm^{-1} (C=O/Phth); ^1H NMR (400 MHz, $CDCl_3$): δ 7.88 (m, 2 H, Phth), 7.75 (m, 2 H, Phth), 5.86 (dd, 1 H, $J_{2,3}$ 10.4, $J_{3,4}$ 9.2 Hz, H-3), 5.66 (d, 1 H, $J_{1,2}$ 8.4 Hz, H-1), 5.20 (dd, 1 H, $J_{3,4}$ 9.2, $J_{4,5}$ 10.0 Hz, H-4), 4.31 (dd, 1 H, $J_{5,6}$ 4.8, $J_{6,6'}$ 12.4 Hz, H-6), 4.29 (dd, 1 H, $J_{1,2}$ 8.4, $J_{2,3}$ 10.4 Hz, H-2), 4.21 (dd, 1 H, $J_{5,6'}$ 2.4, $J_{6,6'}$ 12.4 Hz, H-6'), 3.96 (m, 1 H, H-5), 3.66 (s, OH), 2.13, 2.06, 1.88 (3 s, 9 H, 3 x OAc); ^{13}C NMR (100 MHz, $CDCl_3$): δ 171.06, 170.34, 169.79 (5 x C=O), 134.59, 123.88 (Ph), 92.84 (C-1), 72.24 (C-5), 70.70 (C-3), 69.10 (C-4), 62.27 (C-6), 56.24 (C-2), 20.98, 20.85, 20.67 (3 x acetyl CH_3); Anal. Calcd for $C_{20}H_{21}O_{10}N$: C, 55.17; H, 4.86; N, 3.22. Found: C, 55.43; H, 5.19; N, 3.04.

Tri-*O*-acetyl-2-deoxy-2-tetrachlorophthalimido-β-D-glucopyranose (**4d**)

Procedure a: This was synthesized analogously to **4a**. The following amounts of the substrates were used: **1d** (0.3 g, 0.49 mmol), ethylenediamine (0.038 mL, 0.59 mmol), tetrahydrofuran (13 mL) and glacial acetic acid (38 µL, 0.68 mmol). Column chromathography (solvent B) gave **4d** (0.12 g, white powder, 36%): mp 189 °C; $[\alpha]_D^{20}$ +69 (c 0.5 $CHCl_3$); R_f 0.32 (solvent B); IR: ν 3521 (O-H), 1750, 1785 (C=O/OAc), 1722 cm^{-1} (C=O/TCP); ^1H NMR (400 MHz, $CDCl_3$): δ 5.77 (dd, 1 H, $J_{2,3}$ 10.4, $J_{3,4}$ 9.2 Hz, H-3), 5.63 (d, 1 H, $J_{1,2}$ 8,4 Hz, H-1), 5.20 (dd, 1H, $J_{3,4}$ 9.2, $J_{4,5}$ 10.0 Hz, H-4), 4.26 (dd, 1 H, $J_{1,2}$ 8.4, $J_{2,3}$ 10.4 Hz, H-2), 4.30 (dd, 1 H, $J_{5,6}$ 4.8, $J_{6,6'}$ 12.4 Hz, H-6), 4.20 (dd, 1 H, $J_{5,6'}$ 2.4, $J_{6,6'}$ 12.4 Hz, H-6'), 3.90 (m, 1 H, H-5), 3.28 (d, OH), 2.13, 2.05, 1.91 (3 s, 9 H, 3 x OAc); ^{13}C NMR (100 MHz, $CDCl_3$): δ 170.95, 170.72, 169.64 (5 x C=O), 130.29 (Ph), 92.50 (C-1), 72.36 (C-5),

70.79 (C-3), 68.74 (C-4), 62.11 (C-6), 56.89 (C-2), 21.01, 20.83, 20.74 (3 x acetyl CH_3); Anal. Calcd for $C_{20}H_{17}O_{10}NCl_4$: C, 41.91; H, 2.99; N, 2.44. Found: C, 41.93; H, 2.94; N, 2.45.

Procedure b: A solution of **2d** (0.8 g, 1.3 mmol) in acetone (8 mL), protected from light, was stirred at rt for 5 min. Then a suspension of Ag_2CO_3 (0.65 mmol) in H_2O (4 mL) was added and stirring was continued for 2 h. The reaction was monitored by TLC (solvent C). After filtration of the silver salt, the mixture was diluted with AcOEt (50 mL) and washed with H_2O (20 mL), satd aq NaCl (20 mL), satd aq $NaHCO_3$ (2 x 20 mL) and H_2O (20 mL), dried over $MgSO_4$ and concentrated. Silica gel column chromatography (solvent D) of the crude product afforded **4d** (0.54 g, white powder, 73%).

Procedure c: A solution of **3d** (0.58 g, 0.98 mmol) in acetone (8 mL), protected from light was stirred at rt for 5 min. Then a suspension of Ag_2CO_3 (1.1 mmol) in H_2O (4 mL) was added and stirring was continued for 2 h. The reaction was monitored by TLC (solvent C). After filtration of the silver salt, the mixture was diluted with AcOEt (50 mL) and washed with H_2O (20 mL), satd aq NaCl (20 mL), satd aq $NaHCO_3$ (2 x 20 mL) and H_2O (20 mL), dried over $MgSO_4$ and concentrated. This gave a pale foam, which was crystallized from Et_2O/petroleum ether to afford **4d**. Additional portion of **4d** was obtained after column chromatography of the residue (solvent D) (0.40 g, 72%).

N-Protected 3,4,6-tri-*O*-acetyl-2-deoxy-2-amino-D-glucopyranosyl (*N*-phenyl)trifluoro-acetimidates **5a–d**

General procedure:

The respective *N*-protected 3,4,6-tri-*O*-acetyl-D-glucosamines **4a-d** (1 mmol) were dissolved in anhydrous CH_2Cl_2 (3 mL). Then, K_2CO_3 (2 mmol) and (*N*-phenyl)-2,2,2-trifluoroacetimi-doyl chloride (2 mmol) were added. The mixture was stirred at rt and monitored by TLC (solvent A). After stirring for 6-48 h the mixture was diluted with $CHCl_3$ (80 mL), filtered and

concentrated. The residue was chromatographed on silica gel.

3,4,6-Tri-*O*-acetyl-2-deoxy-2-trifluoroacetamido-α,β-D-glucopyranosyl (*N*-phenyl)trifluoroacetimidate (**5a**)

Reaction of **4a** (0.44 g, 1.1 mmol) with (*N*-phenyl)-2,2,2-trifluoroacetimidoyl chloride (0.46 g, 2.2 mmol), K_2CO_3 (0.30 g, 0.22 mmol) in CH_2Cl_2 (3 mL), followed by column chromatography (solvent B) gave first **5aβ** (0.32 g, syrup, 46%): R_f 0.6 (solvent A); ^{1}H NMR (400 MHz, $CDCl_3$): δ 7.38 (d, 1 H, NH), 7.30 (t, 2 H, NPh), 7.13 (t, 1 H, NPh), 6.75 (d, 2 H, NPh), 5.95 (d, 1 H, $J_{1,2}$ 8.0 Hz, H-1), 5.43 (dd, 1 H, $J_{2,3}$ 10.0, $J_{3,4}$ 9.6 Hz, H-3), 5.13 (dd, 1 H, $J_{3,4}$ 9.6, $J_{4,5}$ 9.2 Hz, H-4), 4.44 (m, 1 H, H-2), 4.27 (dd, 1 H, $J_{5,6}$ 5.2, $J_{6,6'}$ 12.4 Hz, H-6), 4.19 (d, 1 H, $J_{6,6'}$ 12.4 Hz, H-6'), 3.91 (m, 1 H, H-5), 2.07, 2.03, 1.96 (3 s, 9 H, 3 x OAc); ^{13}C NMR (100 MHz, $CDCl_3$): δ 171.93, 170.87, 169.50, 157.57 (C=O), 142.67 (O-C=N), 129.17, 125.06, 119.13 (NPh), 94.23 (C-1), 73.13 (C-5), 71.93 (C-3), 68.30 (C-4), 61.94 (C-6), 53.42 (C-2), 20.82, 20.54, 20. 51 (acetyl CH_3).

Eluted second was **5aα** (0.28 g, syrup, 45%): R_f 0.47 (solvent A); ^{1}H NMR (400 MHz, $CDCl_3$): δ 7.32 (t, 2H, NPh), 7.15 (t, 1H, NPh), 6.89 (bd, 1H, NH), 6.80 (d, 2H, NPh), 6.42 (bs, 1 H, H-1), 5.37 (dd, 1 H, $J_{2,3}$ 10.4, $J_{3,4}$ 9.6 Hz, H-3), 5.26 (t, 1 H, $J_{4,5}$ = $J_{3,4}$ 9.6 Hz, H-4), 4.47 (m, 1 H, H-2), 4.29 (dd, 1 H, $J_{5,6}$ 4.0, $J_{6,6'}$ 12.8 Hz, H-6), 4.12 (d, 1 H, $J_{6,6'}$ 12.8 Hz, H6'), 4.08 (m, 1 H, H-5), 2.11, 2.07 (2 s, 9 H, 3 x OAc); ^{13}C NMR (100 MHz, $CDCl_3$): δ 172.48, 168.88, 168.13, (C=O), 142.64 (O-C=N), 119.35, 125.26, 129.15 (NPh), 92.13 (C-1), 70.40 (C-3), 70.19 (C-5), 67.02 (C-4), 61.45 (C-6), 52.55 (C-2), 20.85, 20.67 (acetyl CH_3).

3,4,6-Tri-*O*-acetyl-2-deoxy-2-(2,2,2-trichloroethoxycarbonylamino)-α,β-D-glucopyranosyl (*N*-phenyl)trifluoroacetimidate (**5b**)

Reaction of **4b** (0.25 g, 0.52 mmol), K_2CO_3 (0.14 g, 1.04 mmol) and (*N*-phenyl)-2,2,2-trifluoroacetimidol chloride (0.22 g, 1.04 mmol) in CH_2Cl_2 (3 mL), followed by column

chromatography (solvent B) gave first **5bβ** (0.17 g, syrup, 49%): R_f 0.68 (solvent A); ^{1}H NMR (400 MHz, $CDCl_3$): δ 7.31 (t, 2 H, NPh), 7.13 (t, 1 H, NPh), 6.82 (d, 2 H, NPh), 5.92 (bs, 1 H, H-1), 5.38 (d, 1 H, NH), 5.30 (dd, 1 H, $J_{2,3}$ 10.0, $J_{3,4}$ 9.6 Hz, H-3), 5.14 (t, 1H, $J_{3,4}$ = $J_{4,5}$ 9.6 Hz, H-4), 4.77 (d, 1 H, CH_2Troc), 4.72 (d, 1 H, CH_2Troc), 4.28 (dd, 1 H, $J_{5,6}$ 4.0, $J_{6,6'}$ 12.4 Hz, H-6), 4.14 (d, 1 H, $J_{6,6'}$ 12.4 Hz, H-6'), 3.98 (m, 1 H, H-2), 3.78 (m, 1 H, H-5), 2.08, 2.05, 2.03 (3 s, 9 H, 3 x OAc); ^{13}C NMR (100 MHz, $CDCl_3$): δ 170.90, 170.83, 169.56, 154.11 (C=O), 143.13 (O-C=N), 129.08, 124.90, 119.40 (NPh), 94.84 (C-1), 74.81 (CH_2Troc), 73.05 (C-5), 71.75 (C-3), 68.14 (C-4), 61.81 (C-6), 55.46 (C-2), 20.90, 20.79, 20.77 (acetyl CH_3).

Eluted second was **5bα** (0.15 g, syrup, 44%): R_f 0.60 (solvent A); ^{1}H NMR (400 MHz, $CDCl_3$): δ 7.31 (t, 2 H, NPh), 7.14 (t, 1H, NPh), 6.80 (d, 2 H, NPh), 6.36 (bs, 1 H, H-1), 5.35 (d, 1 H, NH), 5.33 (dd, 1 H, $J_{3,2}$ 11.2, $J_{3,4}$ 10.4 Hz, H-3), 5.22 (dd, 1 H, $J_{3,4}$ 10.4, $J_{4,5}$ 9.6 Hz, H-4), 4.82 (d, 1 H, CH_2Troc), 4.67 (d, 1 H, CH_2Troc), 4.26 (m, 2 H, H-2, H-6), 4.12 (d, 1 H, $J_{6,6'}$ 11.2 Hz, H-6'), 4.03 (m, 1 H, H-5), 2.10, 2.07, 2.06 (3 s, 9 H, 3 x OAc); ^{13}C NMR (100 MHz, $CDCl_3$): δ 171.32, 170.76, 169.48, 154.43 (C=O), 142.96 (O-C=N), 129.12, 125.08, 119.44 (NPh), 93.96 (C-1), 74.94 (CH_2Troc), 70.30, 67.72 (C-3, C-4, C-5), 61.69 (C-6), 53.81 (C-2), 20.87, 20.76 (acetyl CH_3).

3,4,6-Tri-*O*-acetyl-2-deoxy-2-phthalimido-β-D-glucopyranosyl (*N*-phenyl)trifluoro-acetimidate (**5c**)

Reaction of **4c** (0.19 g, 0.43 mmol), K_2CO_3 (0.12 g, 0.85 mmol) and (*N*-phenyl)-2,2,2-trifluoroacetimidol chloride (0.09 g, 0.43 mmol) in CH_2Cl_2 (3 mL) gave **5c** (0.22 g, syrup, 85%): R_f 0,57 (solvent A); ^{1}H NMR (400 MHz, $CDCl_3$): δ 7.87 (m, 2 H, Phth), 7.77 (m, 2 H, Phth), 7.26 (t, 2 H, NPh), 7.09 (t, 1 H, NPh), 6.72 (d, 2 H, NPh), 6.54 (d, 1 H, $J_{1,2}$ 9.6 Hz, H-1), 5.81 (m, 1 H, H-3), 5.23 (t, 1 H, $J_{3,4}$ 9.6, $J_{4,5}$ 9.6 Hz, H-4), 4.57 (t, 1 H, $J_{1,2}$ = $J_{2,3}$ 9.6 Hz, H-2), 4.31 (dd, 1 H, $J_{5,6}$ 4.0, $J_{6,6'}$ 12.4 Hz, H-6), 4.14 (d, 1 H, $J_{6,6'}$ 12.4 Hz, H-6'), 3.82 (m, 1 H,

H-5), 2.10, 2.03, 1.88 (3 s, 9 H, 3 x OAc); ^{13}C NMR (100 MHz, $CDCl_3$): δ 170.86, 170.28, 169.58 (C=O), 143.00 (O-C=N), 128.91, 124.81, 119.39 (NPh), 134.76, 124.04 (Ph), 92.88 (C-1), 72.92 (C-5), 70.58 (C-3), 68.39 (C-4), 61.61 (C-6), 53.84 (C-2), 20.92, 20.80, 20.63 (acetyl CH_3).

3,4,6-Tri-*O*-acetyl-2-deoxy-2-tetrachlorophthalimido-β-D-glucopyranosyl (*N*-phenyl)trifluoroacetimidate (**5d**)

Reaction of **4d** (0.2 g, 0.35 mmol), K_2CO_3 (0.097 g, 0.70 mmol) and (*N*-phenyl)-2,2,2-trifluoroacetimidol chloride (0.15 g, 0.70 mmol) in CH_2Cl_2 (2 mL), followed by column chromatography (solvent D) gave **5d** (0.18 g, syrup, 68%): R_f 0,63 (solvent A); ^{1}H NMR (400 MHz, $CDCl_3$): δ 7.27 (t, 2 H, NPh), 7.09 (t, 1 H, NPh), 6.76 (d, 2 H, NPh), 6.50 (bs, 1 H, H-1), 5.76 (dd, 1 H, $J_{2,3}$ 9.6, $J_{3,4}$ 10.0 Hz, H-3), 5.25 (dd, 1 H, $J_{3,4}$ 10.0, $J_{4,5}$ 9.2 Hz, H-4), 4.56 (m, 1 H, H-2), 4.32 (dd, 1 H, $J_{5,6}$ 4.4, $J_{6,6'}$ 11.6 Hz, H-6), 4.14 (d, 1 H, $J_{6,6'}$ 11.6 Hz, H-6), 3.90 (m, 1 H, H-5), 2.11, 2.04, 1.92 (3 s, 9 H, 3 x OAc); ^{13}C NMR (100 MHz, $CDCl_3$): δ 170.83, 170.74, 169.50 (C=O), 141.08 (O-C=N), 130.43 (Ph), 128.94, 125.01, 119.50 (NPh), 92.71 (C-1), 72.98 (C-5), 70.80 (C-3), 68.09 (C-4), 61.58 (C-6), 54.60 (C-2), 20.91, 20.78, 20.67 (acetyl CH_3).

Diosgenyl 3,4,6-tri-*O*-acetyl-2-deoxy-2-trifluoroacetamido-β-D-glucopyranoside (**6a**)

From 5a: A mixture of **5a** (0.38 g, 0.66 mmol), diosgenin (0.19 g, 0.45 mmol) and 4 Å molecular sieves (1.5 g) in anhyd CH_2Cl_2 (20 mL) was stirred at rt under N_2 for 30 min. Then TMSOTf (30 μL) was added and stirring at rt was continued. After 20 h the mixture was neutralized by Et_3N, diluted with $CHCl_3$ (50 mL), filtered over the gel layer (MN Kieselgel 60) and washed with H_2O (2 x 10 mL), satd aq $NaHCO_3$ (2 x 10 mL) and again H_2O (10 mL). Then it was dried over $MgSO_4$, filtered and concentrated. Silica gel column chromatography

(solvent E) of the crude product afforded **6a** as a white solid (0.31 g, 85%); results of all analyses are in agreement with those previously reported for the reaction with bromide [4].

Diosgenyl 3,4,6-tri-*O*-acetyl-2-deoxy-2,2,2-trichloroethoxycarbonylamino-β-D-glucopyranoside (**6b**)

*From **2b***: A mixture of diosgenin (0.13 g, 0.31 mmol), AgOTf (0.2 g, 0.72 mmol) and 4Å molecular sieves (1,2 g) in anhydr mixture of Et_2O (10 mL) and CH_2Cl_2 (6 mL) was stirred at rt under N_2 for 10 min. Then a solution of **2b** (0.27 g, 0.50 mmol) in CH_2Cl_2 (8 mL) was allowed to drip on to the reaction mixture. The end of reaction was verified (TLC, solvent A) after stirring for 20 h at rt. Then the mixture was neutralized with Et_3N, diluted with $CHCl_3$, filtered over the gel layer (MN Kieselgel 60), and concentrated. Column chromatography (solvent F) of the crude product afforded **6b** as a white solid (0.27 g, 98%); results of all analyses are in agreement with those previously reported for the reaction with chloride [1].

*From **5b***: A mixture of **5b** (0.26 g, 0.4 mmol), diosgenin (0.11 g, 0.27 mmol) and 4 Å molecular sieves (1 g) in anhyd CH_2Cl_2 (15 mL) was stirred at rt under N_2 for 30 min. Then TMSOTf (10 μL) was added and stirring at rt was continued. After 20 h the mixture was neutralized by Et_3N, diluted with $CHCl_3$ (50 mL), filtered over the gel layer (MN Kieselgel 60) and washed with H_2O (2 x 10 mL), satd aq $NaHCO_3$ (2 x 10 mL) and again H_2O (10 mL). Then it was dried over $MgSO_4$, filtered and concentrated. Silica gel column chromatography (solvent E) of the crude product afforded **6b** as a white solid (0.19 g, 81%); results of all analyses are in agreement with previously reported for the reaction with chloride [1].

Diosgenyl 3,4,6-tri-*O*-acetyl-2-deoxy-2-phthalimido-β-D-glucopyranoside (**6c**)

*From **2c***: A mixture of diosgenin (0.28 g, 0.67 mmol) and 4Å molecular sieves (2.0 g) in anhyd CH_2Cl_2 (20 mL) was stirred at rt under N_2 for 10 min. Then AgOTf (0.45 g, 1.63

mmol) and *s*-collidine (0.35 mL, 2.65 mmol) were added and stirring was continued for **10** min. Then a solution of **2c** (0.52 g, 1.12 mmol) in CH_2Cl_2 (10 mL) was allowed to drip on to the reaction mixture. After stirring for 20 h at rt, the mixture was filtered and concentrated. Precipitation with MeOH and silica gel column chromatography of the filtrate (solvent F) afforded **6c** as a white solid (0.5 g, 90%); results of all analyses are in agreement with previously reported for the reaction with chloride [1].

<u>*From 5c*:</u> A mixture of **5c** (0.22 g, 0.37 mmol), diosgenin (0.10 g, 0.24 mmol), and 4Å molecular sieves (0.8 g) in anhyd CH_2Cl_2 (10 mL) was stirred at room temperature under N_2, for 30 min. Then TMSOTf (10 μL) was added and stirring was continued. After 20 h, the mixture was neutralized by Et_3N, diluted with $CHCl_3$ (50 mL), filtered over the gel layer (MN Kieselgel 60) and washed with H_2O (2 x 10 mL), satd aq $NaHCO_3$ (2 x 10 mL) and again H_2O (10 mL). Then it was dried over $MgSO_4$ and concentrated. Precipitation with MeOH and silica gel column chromatography (solvent F) of the filtrate afforded **6c** as a white solid (0.17 g, 83%): results of all analyses are in agreement with previously reported for the reaction with chloride [1].

Diosgenyl 3,4,6-tri-*O*-acetyl-2-deoxy-2-tetrachlorophthalimido-β-D-glucopyranoside (**6d**)

<u>*From 5d*:</u> A mixture of **5d** (0.16 g, 0.22 mmol), diosgenin (0.066 g, 0.16 mmol), and 4Å molecular sieves (0.5 g) in anhyd CH_2Cl_2 (10 mL) was stirred at room temperature under N_2, for 30 min. Then TMSOTf (10 μL) was added and stirring was continued. After 20 h, the mixture was neutralized by Et_3N, diluted with $CHCl_3$ (50 mL), filtered over the gel layer (MN Kieselgel 60) and washed with H_2O (2 x 10 mL), satd aq $NaHCO_3$ (2 x 10 mL) and again H_2O (10 mL). Then it was dried over $MgSO_4$ and concentrated. Addition of MeOH caused the

precipitation of **6d** as a white solid (0.08 g, 52%): results of all analyses are in agreement with previously reported for the reaction with bromide [4].

General procedure for *N*-alkylation of **7**

The appropriate amount of diosgenyl 2-amino-2-deoxy-β-D-glucopyranoside (**7**) [1] was dissolved in $CHCl_3$/MeOH (1:1, v/v) and 1.2-fold excess of suitable aldehyde was added. The mixture was stirred for 0.5 h at rt. Then 2-fold excess of $NaBH_3CN$ was added and stirring was continued for 1-3 hours. The end of reaction was detected by TLC (solvents G-J). Then the mixtures were evaporated to dryness. The residue was chromatographed on silica gel.

Diosgenyl 2-deoxy-2-dimethylamino-β-D-glucopyranoside (**8**)

Reaction of **7** (0.1 g, 0.17 mmol) with formaldehyde (5.8 µl, 0.2 mmol) and $NaBH_3CN$ (21.4 mg, 0.34 mmol) in $CHCl_3$/MeOH (8 ml) followed by column chromatography (solvent G) gave **8** (49 mg, 78%): mp 235 ^{o}C; $[\alpha]_D^{20}$ -56° (*c* 0.5, $CHCl_3$/MeOH, 1:1); R_f 0,46 (solvent G); ^{1}H NMR (400 MHz): δ 4.70 (d, 1 H, $J_{1,2}$ 8.3 Hz, H-1), 3.83 (dd, 1 H, $J_{5,6'}$ 2.9, $J_{6,6'}$ 12.0 Hz, H-6'), 3.70 (dd, 1 H, $J_{5,6}$ 5.4, $J_{6,6'}$ 12.0 Hz, H-6), 3.46 (dd, 1 H, $J_{2,3}$ 8.8, $J_{3,4}$ 10.2 Hz, H-3), 3.37 (dd, $J_{3,4}$ 10.2, $J_{4,5}$ 9.3 Hz, H-4), 3.33 (m, 1 H, H-5), 2.52 (s, 6 H, CH_3), 2.38 (dd, 1 H, $J_{1,2}$ 8.3, $J_{2,3}$ 8.8 Hz, H-2); diosgenyl protons: 5.32 (d, C_6-H), 4.40 (dd, C_{16}-H), 3.61 (m, C_{26}-H_e), 3.32 (t, C_{26}-H_a), 2.31 and 2.00 (m, C_4-2H), 1.20 (s, $CH_{3(19)}$), 0.98 (d, $CH_{3(21)}$), 0.80 (s, $CH_{3(18)}$), 0.79 (d, $CH_{3(27)}$); ^{13}C NMR (100 MHz): δ 98.86 (C-1), 78.28 (C-3), 77.23 (C-5), 75.76 (C-4), 66.51 (C-6), 61.80 (C-2), 49.90 (CH_3); diosgenyl carbons: 140.07 (C-5), 121.42 (C-6), 109.28 (C-22), 80.71 (C-16), 77.55 (C-3), 71.00 (C-26), 61.49 (C-17), 56.21 (C-14), 40.95 (C-20), 39.95 (C-13), 39.40 (C-12), 38.96 (C-4), 36.86 (C-1), 36.52 (C-10), 20.48 (C-11), 18.77 (C-19), 16.38 (C-27), 15.69 (C-18), 13.75 (C-21); MALDI-TOF-MS: *m/z* 627.00 $(M+ Na)^+$.

Diosgenyl 2-deoxy-2-ethylamino- (**9**) and -2-diethylamino-β-D-glucopyranoside (**10**)

Reaction of **7** (60 mg, 0.1 mmol) with acetaldehyde (9.2 µl, 0.12 mmol) and $NaBH_3CN$ (12.6 mg, 0.2 mmol) in $CHCl_3$/MeOH (5 ml) followed by column chromatography (solvent H) gave two products. The first was **9** (23.8 mg, 38%): mp 194 °C; $[\alpha]^{D}_{20}$ -43° (*c* 0.5, $CHCl_3$/MeOH); R_f 0,47 (solvent G); ^{1}H NMR (400 MHz): δ 4.46 (d, 1 H, $J_{1,2}$ 7.8 Hz, H-1), 3.83 (dd, 1 H, $J_{5,6'}$ 2.9, $J_{6,6'}$ 12.2 Hz, H-6'), 3.71 (dd, 1 H, $J_{5,6}$ 4.9, $J_{6,6'}$ 12.2 Hz, H-6), 3.38-3.28 (m, 2 H, H-3, H-4), 3.25 (m, H-5), 3.30 (m, 1 H, NCH_a), 2.80 (m, 1 H, NCH_b), 2.45 (dd, 1 H, $J_{1,2}$ 7.8, $J_{2,3}$ 8,8 Hz, H-2), 1.14 (t, 3 H, CH_3); diosgenyl protons: 5.36 (d, C_6-H), 4.40 (dd, C_{16}-H), 3.58 (m, C_{26}-H_e), 3.45 (m, C_{26}-H_a), 2.40 and 2.24 (m, C_4-2H), 1,15 (s, $CH_{3(19)}$), 0.96 (d, $CH_{3(21)}$), 0.80 (s, $CH_{3(18)}$), 0.79 (d, $CH_{3(27)}$); ^{13}C NMR (100 MHz): δ 98.41 (C-1), 79.21 (C-5), 76.71 (C-3), 72.81 (C-4), 61.28 (C-6), 50.33 (C-2), 42.56 (NCH_2), 11.76 (CH_3); diosgenyl carbons: 140.24 (C-5), 122.09 (C-6), 109.67 (C-22), 81.11 (C-16), 72.81 (C-3), 70.84 (C-26), 61.96 (C-17), 56.62 (C-14), 41.77 (C-20), 40.36 (C-13), 39.79 (C-12), 38.62 (C-4), 37.24 (C-1), 36.88 (C-10), 20.90 (C-11), 19.08 (C-19), 16.72 (C-27), 16.03 (C-18), 14.09 (C-21); MALDI-TOF-MS: *m/z* 627.00 $(M+ Na)^+$.

The second was **10** (19.4 mg, 49%): mp 196 °C; $[\alpha]_D^{20}$ -39° (*c* 0.5, $CHCl_3$/MeOH); R_f 0,85 (solvent G); ^{1}H NMR (400 MHz): δ 4.72 (d, 1 H, $J_{1,2}$ 8.4 Hz, H-1), 3.83 (dd, 1 H, $J_{5,6'}$ 2.9, $J_{6,6'}$ 11.7 Hz, H-6'), 3.72 (dd, 1 H, $J_{5,6}$ 4.9, $J_{6,6'}$ 11.7 Hz, H-6), 3.44 (m, 1 H, H-4), 3.34 (m, 1 H, H-3), 3.26 (m, 1 H, H-5), 2.84 (m, 4 H, 2 x NCH_a, 2 x NCH_b), 2.62 (dd, 1 H, $J_{1,2}$ 8.4, $J_{2,3}$ 9,6 Hz, H-2), 1.10 (t, 6 H, 2 x CH_3); diosgenyl protons: 5.38 (d, C_6-H), 4.40 (dd, C_{16}-H), 3.58 (m, C_{26}-H_e), 2.38 and 2.20 (2 x m, C_4-2H), 0.98 (d, $CH_{3(21)}$), 0.80 (s, $CH_{3\ (18)}$), 0.79 (d, $CH_{3\ (27)}$); ^{13}C NMR (100 MHz): δ 99.47 (C-1), 78.46 (C-3), 77.23 (C-5), 75.87 (C-4), 66.51 (C-2), 64.62 (C-6), 49.89 (NCH_2), 14.31 (CH_3); diosgenyl carbons: 140.09 (C-5), 121.39 (C-6), 109.28 (C-22), 80.71 (C-16), 78.46 (C-3), 71.32 (C-26), 61.51 (C-17), 56.21 (C-14), 41.33 (C-20), 39.95 (C-13), 39.39 (C-12), 38.91 (C-4), 36.85 (C-1), 36.51 (C-10), 20.48 (C-11), 18.79 (C-19), 16.39 (C-27), 15.70 (C-18), 13.75 (C-21), MALDI-TOF-MS: *m/z* 655.00

$(M+Na)^+$.

Diosgenyl 2-deoxy-2-propylamino- (**11**) and -2-dipropylamino-β-D-glucopyranoside (**12**)

Reaction of **7** (200 mg, 0.35 mmol) with propionaldehyde (30 µl, 0.42 mmol) and $NaBH_3CN$ (43.8 mg, 0.69 mmol) in $CHCl_3$/MeOH (15 ml) followed by column chromatography (solvent I) gave two products. The first was **11** (103 mg, 48%): mp 180 °C; $[\alpha]^D_{20}$ -47° (*c* 0,5 $CHCl_3$/MeOH); R_f 0,34 (solvent I); ^{1}H NMR (400 MHz): *δ* 4.44 (d, 1 H, $J_{1,2}$ 8.0 Hz, H-1), 3.85 (dd, 1 H, $J_{5,6'}$ 3.2, $J_{6,6'}$ 11.8 Hz, H-6'), 3.75 (dd, 1 H, $J_{5,6}$ 5.0, $J_{6,6'}$ 11.8 Hz, H-6), 3.47 (t, 1 H, $J_{3,4}$ 9.2, $J_{4,5}$ 9.6 Hz, H-4), 3.35 (t, 1 H, $J_{2,3}$ 9.8, $J_{3,4}$ 9.2 Hz, H-3), 3.28 (m, 1 H, H-5), 2.91 (m, 1 H, NCH_a), 2.70 (m, 1 H, NCH_b), 2.42 (dd, 1 H, $J_{1,2}$ 8.0, $J_{2,3}$ 9.6 Hz, H-2), 1.54 (m, 2 H, CH_2), 0.96 (t, 3 H, CH_3); diosgenyl protons: 5.38 (d, C_6-H), 4.40 (dd, C_{16}-H), 3.59 (m, C_{26}-H_e), 2.31 and 2.00 (m, C_4-2H), 1.05 (s, $CH_{3(19)}$), 0.81 (s, $CH_{3(18)}$), 0.78 (d, $CH_{3(27)}$); ^{13}C NMR (100 MHz): δ 101.87 (C-1), 78.84 (C-5), 75.84 (C-3), 74.94, (C-4), 62.92 (C-6), 61.82 (C-2), 49.88 (NCH_2), 22.64 (CH_2), 10.93 (CH_3); diosgenyl carbons: 140.12 (C-5), 121.38 (C-6), 109.27 (C-22), 80.71 (C-16), 70.56 (C-26), 61.51 (C-17), 56.21 (C-14), 41.34 (C-20), 39.95 (C-13), 39.41 (C-12), 38.52 (C-4), 36.90 (C-1), 36.51 (C-10), 20.49 (C-11), 18.79 (C-19), 16.41 (C-27), 15.71 (C-18), 13.77 (C-21), MALDI-TOF-MS: *m/z* 619.00 $(M+H)^+$.

The second was **12** (58 mg, 45%): mp 171 °C; $[\alpha]^D_{20}$ -40° (c 0.5, $CHCl_3$/MeOH); R_f 0,60 (solvent I); ^{1}H NMR (400 MHz): *δ* 4.38 (d, 1 H, $J_{1,2}$ 8.4 Hz, H-1), 3.68 (t, 1 H, $J_{3,4}$ 9.2, $J_{4,5}$ 9.6 Hz, H-4), 3.46 (dd, 1 H, $J_{5,6}$ 4.8, $J_{6,6'}$ 12.4 Hz, H-6), 3.50-3.35 (m, 3 H, H-3, H-5 and H-6'), 2.50 (dd, 1 H, $J_{1,2}$ 8.4 $J_{2,3}$ 10.4 Hz, H-2), 2.32 (m, 2 H, 2 x NCH_a), 2.18 (m, 2 H, 2 x NCH_b), 1.60 (m, 4 H, 2 x CH_2), 1.08 (t, 6 H, 2 x CH_3); diosgenyl protons: 5.37 (d, C_6-H), 4.40 (dd, C_{16}-H), 3.61 (m, C_{26}-H_e), 3.32 (t, C_{26}-H_a), 2.00 (m, C_4-2H), 0.97 (d, $CH_{3(21)}$), 0.80 (s, $CH_{3(18)}$), 0.79 (d, $CH_{3(27)}$); ^{13}C NMR (100 MHz): δ 103.32 (C-1), 79.42, 75.84, 74.99, (C-3, C-4, C-5),

62.33 (C-6), 61.51 (C-2), 50.29 (NCH_2), 32.07, 22.68 (CH_2), 11.92 (CH_3); diosgenyl carbons: 140.38 (C-5), 121.49 (C-6), 109.52 (C-22), 81.02 (C-16), 70.26 (C-26), 61.51 (C-17), 56.70 (C-14), 41.83 (C-20), 39.96 (C-13), 39.52 (C-12), 39.12 (C-4), 37.39 (C-1), 37.06 (C-10), 21.06 (C-11), 19.61 (C-19), 17.36 (C-27), 16.52 (C-18), 14.76 (C-21); MALDI-TOF-MS: *m/z* 661.00 $(M+ H)^+$.

Diosgenyl 2-deoxy-2-dibutylamino-β-D-glucopyranoside (**13**)

Reaction of **7** (170 mg, 0.3 mmol) with butyraldehyde (32.4 µl, 0.36 mmol) and $NaBH_3CN$ (37.7 mg, 0.58 mmol) in $CHCl_3$/MeOH (10 ml) followed by column chromatography (solvent J) yielded **13** (51 mg, 42%): R_f 0,61 (solvent J); ^{1}H NMR (400 MHz): δ 4.42 (d, 1 H, $J_{1,2}$ 8.0 Hz, H-1), 3.82 (dd, 1 H, $J_{5,6'}$ 2.9, $J_{6,6'}$ 11.6 Hz, H-6'), 3.76 (dd, 1 H, $J_{5,6}$ 4.8, $J_{6,6'}$ 11.6 Hz, H-6), 3.51 (m, 1 H, H-4), 3.42 (m, 1 H, H-3), 3.30 (m, 1 H, H-5), 2.42 (m, 4 H, NCH_a, NCH_b), 2.40 (dd, 1 H, $J_{1,2}$ 8.0, $J_{2,3}$ 9.6 Hz, H-2), 1.60 (m, 4 H, 2 x CH_2), 1.35 (m, 4 H, 2 x CH_2), 0.92 (t, 6 H, 2 x CH_3); diosgenyl protons: 5.36 (d, C_6-H), 4.40 (dd, C_{16}-H), 3.40 (m, C_{26}-H_e), 2.32 and 2.00 (m, C_4-2H), 1.02 (s, $CH_{3(19)}$), 0.98 (d, $CH_{3(21)}$), 0.80 (s, $CH_{3(18)}$), 0.78 (d, $CH_{3(27)}$); MALDI-TOF-MS: *m/z* 690.00 $(M+ H)^+$.

Diosgenyl 2-deoxy-2-diisobutylamino-β-D-glucopyranoside (**14**)

Reaction of **7** (170 mg, 0.3 mmol) with isovaleric aldehyde (38.76 µl, 0.36 mmol) and $NaBH_3CN$ (37.7 mg, 0.58 mmol) in $CHCl_3$/MeOH (7ml) followed by column chromatography (solvent I) yielded **14** (48.6 mg, 38%): R_f 0,56 (solvent I); ^{1}H NMR (400 MHz): δ 4.50 (d, 1 H, $J_{1,2}$ 8.0 Hz, H-1), 3.86 (dd, 1 H, $J_{5,6'}$ 2.9, $J_{6,6'}$ 11.8 Hz, H-6'), 3.80 (dd, 1 H, $J_{5,6}$ 4.9, $J_{6,6'}$ 11.8 Hz, H-6), 3.56 (m, 1 H, H-4), 3.48 (m, 1 H, H-3), 3.36 (m, 1 H, H-5), 2.92 (m, 4 H, 2 x NCH_a, 2 x NCH_b), 2.46 (m, 1 H, H-2), 1.79 (m, 4 H, 2 x CH_2), 1.30 (m, 2 H, 2 x CH), 0.96 (d, 12 H, 4 x CH_3); diosgenyl protons: 5.32 (m, C_6-H), 4.30 (dd, C_{16}-H), 3.59 (m, C_{26}-H_e), 3.32 (m, C_{26}-H_a), 2.30 and 2.00 (m, C_4-2H), 1.20 (s, $CH_{3\,(19)}$), 0.98 (d, $CH_{3\,(21)}$), 0.80

(s,$CH_{3\,(18)}$), 0.76 (d, $CH_{3\,(27)}$); MALDI-TOF-MS: *m/z* 717.00 $(M+H)^+$.

Diosgenyl 2-deoxy-2-pentylamino-β-D-glucopyranoside (**15**)

Reaction of **7** (170 mg, 0.3 mmol) with valeric aldehyde (38.3 µl, 0.36 mmol) and $NaBH_3CN$ (37.7 mg, 0.58 mmol) in $CHCl_3$/MeOH (10ml) followed by column chromatography (solvent J) gave **15** (40 mg, 21%): R_f 0,56 (solvent J); IR: ν 3373-3473 (O-H and N-H), 2847- 2932 (C-H), 1593 cm^{-1} (N-H); ^1H NMR (400 MHz): δ 4.55 (d, 1 H, $J_{1,2}$ 8.0 Hz, H-1), 3.83 (dd, 1 H, $J_{5,6'}$ 2.8, $J_{6,6'}$ 12.0 Hz, H-6'), 3.70 (dd, 1 H, $J_{5,6}$ 4.8, $J_{6,6'}$ 12.0 Hz, H-6), 3.45 (m, 1 H, H-4), 3.36 (m, 1 H, H-3), 3.24 (ddd, 1 H, $J_{5,6'}$ 2.8, $J_{5,6}$ 4.8, $J_{4,5}$ 9.6 Hz, H-5), 3.50 (m, 1 H, H-2), 2.20 (m, 2 H, NCH_a, NCH_b), 1.59 (m, 2 H, CH_2), 1.20 (m, 4 H, 2 x CH_2), 1.00 (t, 3 H, CH_3); diosgenyl protons: 5.32 (m, C_6-H), 4.30 (dd, C_{16}-H), 3.59 (m, C_{26}-H_e), 3.32 (m, C_{26}-H_a), 2.30 and 2.00 (m, C_4-2H), 1.20 (s, $CH_{3(19)}$), 0.98 (d, $CH_{3(21)}$), 0.80 (s,$CH_{3(18)}$), 0.76 (d, $CH_{3(27)}$); MALDI-TOF-MS: *m/z* 647.00 $(M+H)^+$.

Diosgenyl 2-deoxy-2-hexylamino- (**16**) and -2-dihexylamino-β-D-glucopyranoside (**17**)

Reaction of **7** (100 mg, 0.17 mmol) with hexanoic aldehyde (25.5 µl, 0.21 mmol) and $NaBH_3CN$ (21.4 mg, 0.33 mmol) in $CHCl_3$/MeOH (8 ml) followed by column chromatography (solvent I) yielded two compounds. The first was **16** (60 mg, 52%): mp 175 ^oC, R_f 0,40 (solvent I); ^1H NMR (400 MHz): δ 4.69 (d, 1 H, $J_{1,2}$ 8.4 Hz, H-1), 3.89 (dd, 1 H, $J_{5,6'}$ 2.4, $J_{6,6'}$ 11.6 Hz, H-6'), 3.76 (dd, 1 H, $J_{5,6}$ 5.2, $J_{6,6'}$ 11.6 Hz, H-6), 3.59- 3.41 (m, 2 H, H-4, H-5), 3.38 (dd, 1 H, $J_{2,3}$ 10.4, $J_{3,4}$ 9.2 Hz, H-3), 2.65 (m, 2 H, NCH_a, NCH_b), 2.51 (dd, 1 H, $J_{1,2}$ 8.4, $J_{2,3}$ 10.4 Hz, H-2), 1.60 (m, 2 H, CH_2), 1.30 (m, 6 H, 3 x CH_2), 0.88 (t, 3 H, CH_3); diosgenyl protons: 5.36 (d, C_6-H), 4.40 (dd, C_{16}-H), 3.64 (m, C_{26}-H_e), 2.32 and 2.00 (m, C_4-2H), 1.20 (s, $CH_{3(19)}$), 0.97 (d, $CH_{3(21)}$), 0.79 (s,$CH_{3(18)}$), 0.78 (d, $CH_{3(27)}$); MALDI-TOF-MS: *m/z* 659.94 $(M)^+$, 661.00 $(M+H)^+$.

The second was **17** (16,9 mg, 22%): mp 180 ^{o}C, R_f 0,53 (solvent I); ^{1}H NMR (400 MHz): δ 4.50 (d, 1 H, $J_{1,2}$ 8.4 Hz, H-1), 3.86 (dd, 1 H, $J_{5,6'}$ 3.0, $J_{6,6'}$ 11.6 Hz, H-6'), 3.80 (dd, 1 H, $J_{5,6}$ 4.4, $J_{6,6'}$ 11.6 Hz, H-6), 3.56 (m, 1 H, H-4), 3.36 (m, 1 H, H-5), 3.48 (dd, 1 H, $J_{2,3}$ 9.6, $J_{3,4}$ 10.8 Hz, H-3), 2.92 (m, 4 H, 2 x NCH_a, 2 x NCH_b), 2.46 (dd, 1 H, $J_{1,2}$ 8.4, $J_{2,3}$ 9.6 Hz, H-2), 1.48 (m, 4 H, 2 x CH_2), 1.30 (m, 12 H, 6 x CH_2), 0.89 (t, 6 H, 2 x CH_3); diosgenyl protons: 5.36 (d, C_6-H), 4.40 (dd, C_{16}-H), 3.58 (m, C_{26}-H_e), 3.38 (m, C_{26}-H_a), 2.28 and 2.00 (m, C_4-2H), 1.02 (s, $CH_{3\ (19)}$), 0.98 (d, $CH_{3\ (21)}$), 0.79 (s,$CH_{3(18)}$), 0.78 (d, $CH_{3(27)}$); MALDI-TOFMS: *m/z* 744.0 $(M)^+$, 745.00 $(M+ H)^+$.

Determination of minimum inhibitory concentration

The following microbial strains: *Aspergillus niger* ATCC 16404, *Bacillus subtilis* ATCC 6633, *Candida albicans* ATCC 10231, *Candida tropicalis* PCM 2681, *Enterococcus faecalis* ATCC 29212, *Escherichia coli* ATCC 25922, *Klebsiella pneumoniae* ATCC 13882, *Proteus mirabilis* PCM 543, *Proteus vulgaris* ATCC 13315, *Pseudomonas aeruginosa* ATCC 9027, *Rhodococcus equi* ATCC 6939, *Staphylococcus aureus* ATCC 25923 and *Staphylococcus epidermidis* PCM 2118 were purchased from the Polish Academy of Sciences (Wroclaw, Poland). Reference strains of bacteria were inoculated in Mueller-Hinton II broth (MHB II) (*Sigma-Aldrich, Germany*) 24 h before performing the test and incubated at 37 °C with 150 rpm shaking. Fungal isolates were inoculated in *Sabouraud* 2% glucose broth (*Carl Roth GmbH, Germany*) and cultured for 48 h at 25 °C with 150 rpm shaking before the minimum inhibitory concentration was tested.

Minimum inhibitory concentration (MIC) was determined using a serial dilution method according to the guidelines of Clinical and Laboratory Standards Institute (CLSI). Mueller MHB II and initial inoculums of 5×10^5 CFU/ml were used for tested bacterial strains. For the tested fungi, *Sabouraud* glucose 2% broth and initial inoculums 10^3 CFU/ml were applied.

The microbes were inoculated to polystyrene 96-well plates *(Becton Dickinson)* and exposed to graded concentrations of saponins (range 0.5 – 1024 mg/L). The compounds were dissolved in 10% DMSO in a phosphate buffer. The tests containing bacteria were incubated for 18 h at 37˚C, while the ones with fungi for 48h at 25˚C. MIC was taken as the lowest concentration of the compound at which a noticeable growth was inhibited. The experiments were performed in triplicate on three different days.

References:

1. Bednarczyk, D; Walczewska, A.; Grzywacz, D.; Sikorski, A.; Liberek, B.; Myszka, H. *Carbohydr. Res.* **2013**, *367*, 10-17.
2. Higashi, K.; Nakayama, K.; Soga, T.; Shioya, E.; Uoto, K.; Kusama, T. *Chem. Pharm. Bull.* **1990**, *38*, 3280-3282.
3. Sala, R. F.; MacKinnon, S. L.; Palcic, M. M.; Tanner, M. E. *Carbohydr. Res.* **1998**, *306*, 127-136.
4. Myszka, H.; Bednarczyk, D.; Najder, M.; Kaca, W. *Carbohydr. Res.* **2003**, *338*, 133-141.
